# A commentary on ‘Risk factors for incident venous thromboembolism in patients with renal tumor and inferior vena cava tumor thrombus: a retrospective case–control study’

**DOI:** 10.1097/JS9.0000000000001259

**Published:** 2024-03-18

**Authors:** Aimin Jiang, Le Qu, Peng Luo, Linhui Wang

**Affiliations:** a Department of Urology, Changhai Hospital, Naval Medical University, Shanghai; b Department of Urology, Jinling Hospital, Affiliated Hospital of Medical School, Nanjing University, Nanjing, Jiangsu; c Department of Oncology, Zhujiang Hospital, Southern Medical University, Guangzhou, People’s Republic of China

*Dear Editor*,

Cancer-associated thrombosis, encompassing both arterial and venous thromboembolism (VTE), is a significant contributor to non-cancer mortality among cancer patients^1^. Various risk factors, such as cancer type, stage, treatment, and comorbidities, contribute to the development of VTE, indicating a multifactorial etiology. A recent publication in the *International Journal of Surgery* by Wang *et al*., titled ‘Risk factors for incident venous thromboembolism in patients with renal tumor and inferior vena cava tumor thrombus: a retrospective case–control study’ piqued our interest^2^. The authors addressed a critical gap in the genitourinary-oncology field by identifying preoperative risk factors for VTE in patients with renal tumors and inferior vena cava tumor thrombus (RT-IVCTT). The significance of this research lies in its potential to refine risk assessment and enhance perioperative care for this high-risk population. The retrospective case–control analysis, encompassing 257 patients over a 14-year period, brings to light the critical impact of advanced IVC thrombus, renal sinus fat invasion, IVC wall invasion, significant IVC blockage, and elevated neutrophil levels on the risk of VTE in this patient group. Notably, this research underscored the importance of serum albumin levels as an independent risk factor for VTE, diverging from traditional risk assessments focused solely on tumor location or size. The study’s findings could revolutionize preoperative risk stratification, guiding clinicians towards more personalized VTE management strategies for patients with RT-IVCTT. By highlighting the multifaceted nature of VTE risk in these patients, the authors emphasized the need for a nuanced approach to preoperative planning, potentially improving patient outcomes through targeted risk mitigation efforts. Despite the strengths of this study, several points need further discussion.

First, although the researchers included several preclinical parameters, some important variables that play a role in the occurrence of VTE were not included in the analysis. These omitted factors include the preoperative renal function of patients (creatinine levels and glomerular filtration rate) and patients’ physical fitness (such as Karnofsky’s performance score). Previous reports have found that renal dysfunction is closely associated with a higher risk of both primary and recurrent VTE^3^. Similarly, reduced activity is a significant factor contributing to the increased risk of VTE in cancer patients compared to non-cancer patients^4^. Thus, we suggest that more VTE-relevant characteristics should be enrolled to identify novel risk factors.

Second, it is well-established that various adjuvant medications have the potential to induce thrombosis, as evidenced by numerous case studies demonstrating a correlation between chemotherapy, targeted therapy, and thrombosis^5^. Specifically, the utilization of vascular endothelial growth factor (VEGF) inhibitors, including bevacizumab, as well as VEGF tyrosine kinase receptor inhibitors (such as sorafenib, sunitinib, and pazopanib), has been associated with a heightened risk of venous thrombosis^6^. Nonetheless, the study had a restricted number of patients who underwent neoadjuvant therapy, with treatment rates of 20.6% in the venous thrombosis group and 19.6% in the non-venous thrombosis group, and comprehensive medication details were not disclosed.

Third, there is an inconsistency in the description provided in this work, which may lead to confusion for readers. It is suggested that the description of ‘Except hemoglobin, categorically coded serum albumin was also validated as an independent *risk* factor for VTE’ in the results section be corrected to ‘Except hemoglobin, categorically coded serum albumin was also validated as an independent *protective* factor for VTE’ as indicated by the odds ratios of 0.467 (0.260–0.838, *P*=0.011) and 0.356 (0.170–0.746, *P*=0.006) for higher levels of serum albumin (categorical) in Table 2 for univariable and multivariable analyses, respectively^2^.

In general, this study presents numerous opportunities for further investigation. Subsequent research should aim to confirm the identified risk factors and potentially discover additional predictors of VTE in this specific patient cohort. Furthermore, delving into the mechanistic foundations of the correlation between renal tumors, inferior vena cava involvement, and VTE could yield significant insights. Additionally, assessing the effectiveness of preoperative interventions guided by the identified risk factors may facilitate the creation of tailored and efficient strategies for preventing venous thromboembolism.

## Ethical approval

It is not necessary.

## Consent

Not applicable.

## Sources of funding

None.

## Author contribution

A.J. and L.Q.: Data collection and manuscript preparation; P.L. and L.W.: Study concept or design, critical revision, and submission. A.J., L.Q., and P.L. have contributed equally to this work.

## Conflicts of interest disclosure

The author declares no conflicts of interest.

## Research registration unique identifying number (UIN)

Not applicable.

## Guarantor

Linhui Wang.

## Data availability statement

The datasets generated and/or analyzed during the current study are available from the corresponding author on reasonable request.

## Provenance and peer review

Not applicable.
